# A New Method to Quantify within Dive Foraging Behaviour in Marine Predators

**DOI:** 10.1371/journal.pone.0099329

**Published:** 2014-06-12

**Authors:** Karine Heerah, Mark Hindell, Christophe Guinet, Jean-Benoît Charrassin

**Affiliations:** 1 Laboratoire d'Océanographie et du Climat: Expérimentations et Approches Numériques, CNRS-IRD-MNHN, Paris, France; 2 Institute for Marine and Antarctic Studies, University of Tasmania, Hobart, Australia; 3 Antarctic Climate and Ecosystem Cooperative Research Centre, University of Tasmania, Hobart, Australia; 4 Centre d'Etude Biologique de Chizé, Villiers-en-bois, France; Texas A&M University-Corpus Christi, United States of America

## Abstract

Studies on diving behaviour classically divide a dive into three phases: the descent, bottom and ascent phases, with foraging assumed to occur during the bottom phase. The greater complexity of dive revealed through modern, high resolution data highlights the need to re-assess this approach and to consider a larger number of phases within individual dives. Two southern elephant seals (SES) were fitted with a head mounted Time Depth Recorder (TDR) and an accelerometer from which prey capture attempts were estimated. A Weddell seal was also fitted with a TDR. TDRs for both species recorded depth once per second. We quantified the within dive behaviour using an automated broken stick algorithm identifying the optimal number of segments within each dive. The vertical sinuosity of the segments was used to infer two types of behaviours, with highly sinuous segments indicating "hunting" and less sinuous segments indicating "transiting". Using the broken stick method the seals alternated between "hunting" and "transit" modes with an average of 6±2 and 7±0.02 behavioural phases within each dive for the Weddell seal and SES, respectively. In SES, 77% of prey capture attempts (identified from the acceleration data) occurred in highly sinuous phases (“hunting”) as defined by our new approach. SES spent more time in transit mode within a dive, and hunting mostly occurred during the bottom phase. Conversely the Weddell seal spent more time in hunting mode which also occurred during bottom phase but occurred mostly at shallower depths. Such differences probably reflect different foraging tactics and habitat use. For both species, hunting time differs significantly from bottom time previously used as a proxy for the time spent foraging in a dive. The hunting time defined by our method therefore provides a more accurate fine-scale description of the seals' foraging behaviour.

## Introduction

Predators maximize resource acquisition by adapting their movement patterns and foraging behaviour to the distribution and density of their prey [1]–[3]. In environments where resources are patchily distributed, such as the open ocean, predators need to compensate the costs associated with travel from one patch to another and pursuing a prey with food intake [4]. Thus, predators tend to increase the time spent in the vicinity of recent prey captures by decreasing their displacement speed and increasing their turning frequency [5], [6]. This behaviour, called Area Restricted Search, (ARS) is frequently observed in free ranging predators in the horizontal dimension [7].

For many marine predators, prey capture occurs in the water column where prey are aggregated [8]–[10], making it necessary to also consider the vertical dimension for these species. Identifying feeding events in the vertical dimension (*i.e.* within dives) is still a challenging issue in marine ecology as direct observations are usually impossible. To optimize their foraging strategy when diving, they should decrease their vertical speed and increase the sinuosity of their movements, making what are effectively vertical ARS as indicted on two dimensional dive profiles [11].

Bio-logging devices measure various parameters of free-ranging animal behaviour providing important information on their diving and foraging that are difficult to observe otherwise [12]. Miniaturization, extended battery life and memory size now mean that Time Depth Recorders (TDRs) collect and store data at very high resolutions (one second or less) and for long periods of time (several months) [12], [13], enabling the study of diving behaviour at finer spatial and temporal scales than before [7], [14], [15]. Several foraging metrics (*e.g.* dive duration, dive depth, descent/ascent rate, bottom time, post dive surface interval) can be calculated from TDR data and are used to classify dives into functional categories [7], [16]–[20], but typically they are not systematically associated with direct information on food intake [21]. However, the greater complexity of dives revealed through both high resolution time-depth datasets and three-dimensional diving studies suggest that this method could lead to an over-simplification of diving behaviour [22], [23]. When a seal is spending some time at a particular depth and travelling up and down while at this depth (“wiggles”), it is displaying vertical ARS, and this has been used as an index of foraging activity (not necessarily including prey capture), with several studies providing independent evidence for this in the form of changes in stomach or oesophageal temperature [7], [21], [24]–[26]. More recently, accelerometers measuring body acceleration in up to three dimensions (*i.e.* surge, heave and sway observed in movements such as: stroke and rolling) have provided insights into the functionality of dive types and the details of fine-scale foraging [9], [25]. Stroke frequency has been used as an index of prey pursuit or feeding success [27], [28]. Recent studies have also shown, that for seals, feeding and capture motions are especially visible in the surging axis when using jaw or head accelerometers [25], [29], [30]. Using high resolution dive data in combination with a new approach to detect likely foraging events within a dive can greatly improve what information can be derived from time-depth data.

Southern elephant seals (*Mirounga leonina*, hereafter SES) have a circumpolar distribution and forage extensively across the Southern Ocean [31]. They are associated with important habitats such as the ice edges and continental shelf and feed mainly on fish and squids [32]–[34]. They are also very deep divers, diving up to 2000 meters and performing on average 60 dives per day [16], [35]. Recent studies have focused on SES fine-scale diving behaviour providing more accurate inferences on their foraging activity [7], [25], [36]. However, little is known about SES vertical ARS behaviour, which is more likely to respond directly to prey distribution. A detailed analysis of their vertical excursions during dives in association with prey capture attempts and prey distribution has yet not been conducted.

Weddell seals (*Leptonychotes weddellii*) are the most southerly breeding seal and typically inhabit sea-ice during the whole year [37], [38]. Weddell seals are the second deepest phocid diver in the Southern Ocean after the southern elephant seal, attaining 900 m [39]. They are opportunistic predators feeding mainly on fish, but also on cephalopods and crustaceans, in proportions that vary according to age, location and season [40]. Weddell seal diving and foraging behaviour has been extensively studied during summer in the Ross Sea and the Weddell Sea [29], [41]. However, because Weddell seals are opportunistic predators it is difficult to associate only one type of foraging dive to their overall behaviour [42], [43].

We used high resolution TDR datasets from two SES that travelled to Antarctica during their post-breeding foraging trip and a high resolution TDR dataset covering six winter months from a Weddell seal to develop a new method for identifying the phases within a dive where the seals exhibited foraging behaviour. The concurrent prey capture attempts estimated from high resolution acceleration available for the SES were independently used to validate the method. Our method aimed to: (i) describe the vertical structure and complexity of seal dives, (ii) determine within each dive the parts where likely foraging occurs and (iii) compare this method to classical dive analysis approach.

## Materials and Methods

Fieldwork and data collection were undertaken with approval from the University of Tasmania animal ethics committee (permit A8523), and from IPEV (Institut polaire français Paul Emile Victor) and TAAF (Terres Australes et Antarctiques Françaises) animal ethics committee.

Two adult female SES (length: 266 and 255 cm) were captured at Kerguelen Island (49°20′ S, 70°20′ E) in early November before their post breeding trip. One adult female Weddell seal was captured in February 2008 after its annual moult at Dumont d'Urville (66°40′ S, 140°00 E) (length 230 cm). Similar capture and tagging procedures were adopted for both species. The seals were approached by foot and temporarily restrained with a head bag and an intravenous injection of Zoletil (1∶1 mixture of tiletamine and zolazepam, 0.5 mg.kg^−1^) was administered [44]–[46]. A TDR combined with an accelerometer (TDR Mk 10 X, Wildlife Computers) and a TDR (Mk 10, Wildlife Computers) was head-glued to the SES and to the back of the Weddell seal, respectively, using a two component industrial epoxy (Araldite AW 2101). Seals were observed during recovery from anaesthesia and allowed to enter the water when no longer sedated. The TDRs recorded time and pressure at 1 Hz. Acceleration was recorded in the 3 axis at 16 Hz.

### Fine scale analysis of foraging behaviour

#### 1 Surface offset correction

To account for drift in the pressure transducer accuracy and to identify individual dives, we corrected depths using a customised Zero Offset Correction method. We used a moving window of one hour and considered the modal depth between 20 and −20 meters to represent the true surface (assuming that most of the time in this depth range would represent time on the surface. This depth was then subtracted from all depth values in this interval to provide zero offset corrected depths. Only dives below 15 meters were analysed for the SES, while we defined the Weddell seal's dives as being at least 60 seconds long and four meters deep (60% of all dives) taking into account the accuracy of the pressure transducer (0.5 meters), the size of the seal and sea ice thickness during winter (2.5–3 m, [47]). The frequency distribution of the Weddell seal diving depths was bi-modal, with two groups of dive depth separated at 20 m. Dives <20 m were excluded from further analysis (21% of dives longer than 60 sec.) as they may indicate non-foraging activities [48]. SES performed 3941 and 4254 dives with an average (mean ± SD) of 53±1 (max: 68) and 56±1 (max: 104) dives per day, respectively. The Weddell seal performed 11452 dives deeper than 20 m and longer than one minute with an average of 63±24 (max: 115) dives per day. Standard dive parameters were calculated using classical dive analysis methods [16], [18], [20], dividing each dive into an descent, bottom and ascent phase based on inflection points.

#### 2 Dive analysis with the optimised and automated broken stick method

**Figure 1:**
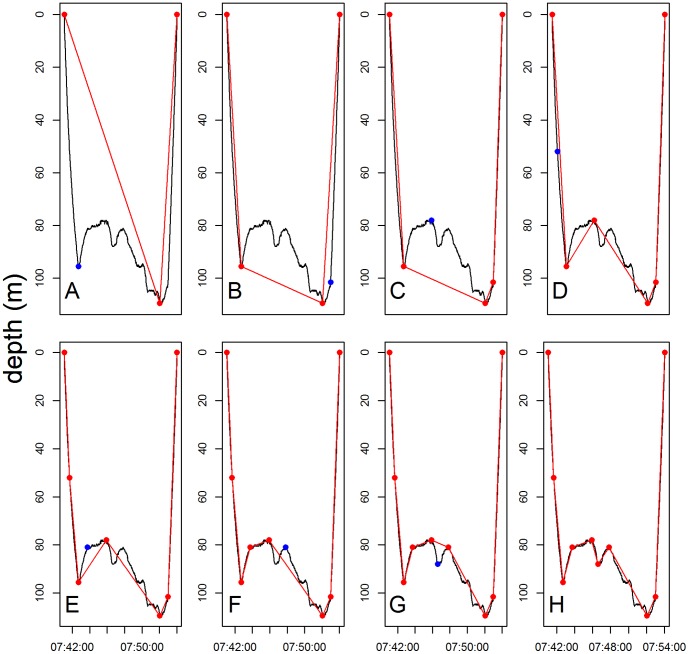
The broken stick algorithm. The iterative process of the broken stick algorithm is presented from panel A to H. The broken stick method iteratively selects the data points (in blue) of maximum difference between the original dive profile (black line) and the dive profile reconstructed by linear interpolation (red lines) between the points selected during the previous iterations (in red). A Weddell seal dive was used as an example for this graph.

As an alternative to the classic three-phases (i.e ascent, bottom and descent) dive analysis (CA) we used a method based on a broken stick algorithm (BS). This method selects the data points where the dive trajectory between two points changes the most rapidly (inflexion points). Any number of points can be chosen depending on the resolution required [49]. We started with three points: (i) surface start point, (ii) maximal depth and (iii) surface end point (Fig.1 A). We then iteratively selected the data points of maximum difference between the original dive profile and the dive profile reconstructed by linear interpolation between the points selected during the previous iterations (Fig.1, Script S1 and Dataset S1).

**Figure 2:**
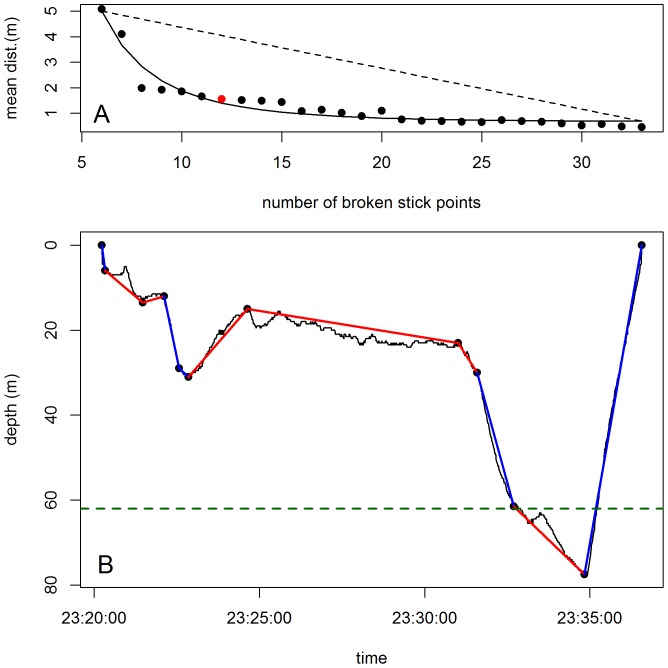
Optimization of the broken stick algorithm. Any number of broken stick points can be chosen depending on the resolution required to describe a dive. A: Mean distance according to the number of broken stick points (from 6 to 33) which are used to describe the dive represented below (B). The mean distance is the average of the differences between each data point of the original profile and the corresponding point of the reconstructed profile obtained by linear interpolation between the broken stick points (from 6 to 33). The inflexion point of the mean distance curve (A, red data point) is determined by calculating the maximal distance between the asymptote curve obtained by fitting a Gompertz model to the mean distance (A, black line) and the linear approximation (A, dashed black line) between its start and end points. B: Original dive profile (B, black line) summarized by the optimal number of broken stick points (B, black data points) as estimated by mean distance represented above (A). The blue lines represent transit segments_BS_ and the red lines represent hunting segments_BS_. The green dashed line represents the depth below which bottom time is calculated with the classical dive analysis method. A Weddell seal dive was used as an example for this graph.

**Figure 3:**
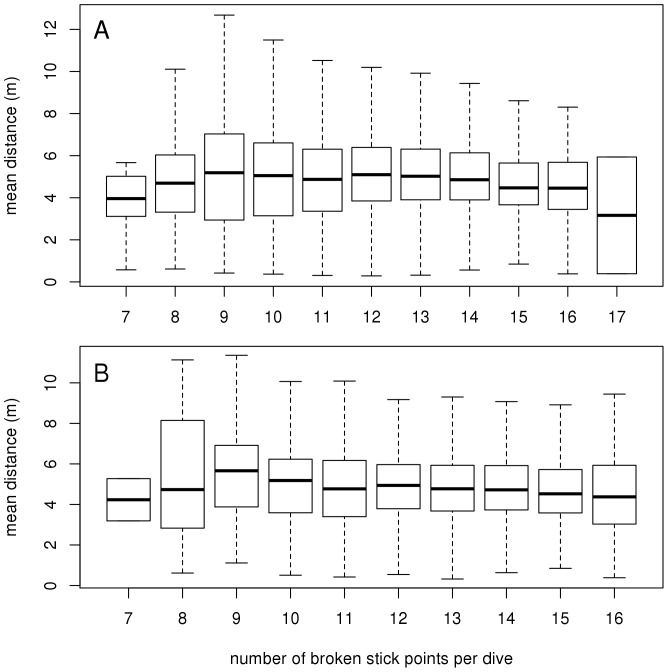
Distribution of the mean distance. Distribution of the mean distance (m) according to the optimal number of broken stick points calculated for each dive for the southern elephant seals (A) and the Weddell seal dataset (B). See figure 2 for calculation of the optimal number of broken stick points.

We then estimated the optimal number of broken stick points (from 6 to 33) that best summarize the dive shape. For this, we calculated a mean distance based on the average of the differences between each data point and its corresponding position on the line between the broken stick points (Fig. 1, averaged depth differences between the black curve and the red lines). The mean distance was calculated for each dive summarised with 6 to 33 broken stick points (Fig. 2 A, Script S1 and Dataset S1). For each dive we plotted the mean distance for a range of broken stick points and we determined the inflexion point of this curve (i.e the point after which the amount of new information explained by increasing the number of segments_BS_ began to decline). To do this in an automated way, each integrated distance curve was smoothed by fitting to a Gompertz model [50]. The inflexion point of this curve was then determined by calculating the maximum distance between the Gompertz curve and the linear approximation between its start and end points (Fig. 2 A, Script S1 and Dataset S1). The number of corresponding broken stick points was then used to optimally describe each dive (Fig. 2, Script S1 and Dataset S1). There was no trend in the relationship between the mean distance and the number of broken stick points per dive (mean ± SD, 5±0.02 m, min: 0.3 m, max: 18 m and 1.2±0.8 m, min: 0.15 m, max: 7.8 m, for the SES and the Weddell seal respectively) (Fig. 3), showing that there is no bias associated with dive complexity.

#### 3 Detection of intensive foraging within dives

Based on the definition of Area Restricted Search (ARS) in the horizontal dimension when animals are at the surface [5], we expected diving predators such as the SES and the Weddell seal to adjust their diving behaviour in order to increase the time spent in a patch of prey, by decreasing their vertical velocity and increasing the vertical sinuosity of their trajectory. Therefore for each segment between two broken stick points (hereafter segment_BS_) we calculated, (i) the vertical descent/ascent rate (in m/s) and (ii) the vertical sinuosity (Script S1 and Dataset S1) adapted from [7] as:
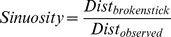
where Dist_broken stick_ is the vertical distance swum between the two broken stick points considered, and the Dist_observed_ is the sum of all the vertical distances from the original dive profile between the two corresponding depth points. Vertical sinuosity ratio (hereafter sinuosity) takes a value of 1 when the individual swims in a straight path during this part of the dive. Any deviation from a straight path decreases the sinuosity ratio toward 0.

**Figure 4:**
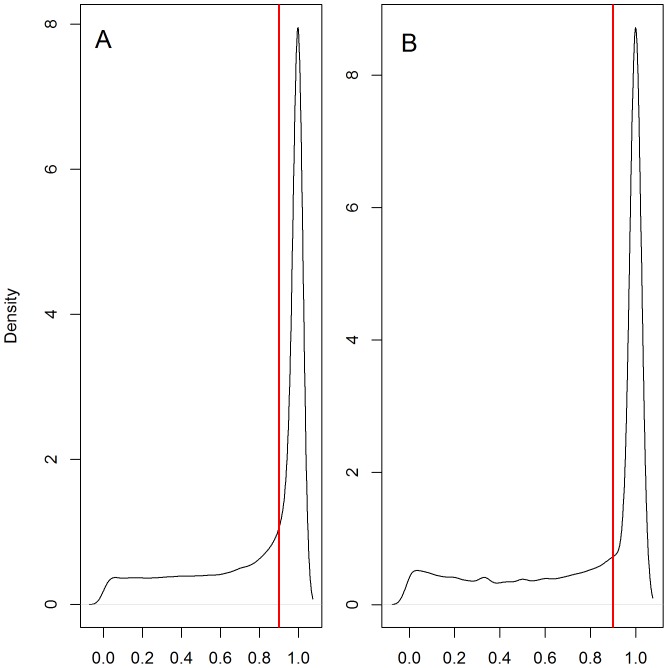
A bimodal behaviour. Density plots representing the distribution of the vertical sinuosity calculated for each broken stick segment from the elephant seal dives (A) and the Weddell seal dives (B). The 0.9 sinuosity threshold represented by the vertical red line was used to discriminate “transit” mode_BS_ versus “hunting” mode_BS_.

The distribution of the sinuosity index of all dive segments_BS_ and for both species was distinctly bi-modal (sinuosity comprised between 0 and 0.9 and sinuosity >0.9, Fig. 4) suggesting two behavioural modes. We used the 0.9 sinuosity threshold to discriminate vertical search mode_BS_ (0< sinuosity >0.9) from directed travel mode_BS_ (0.9≤ sinuosity ≥1) within each dive. Hunting mode_BS_ was characterized by a more sinuous path, possibly indicating intra-patch movements, whereas directed travel mode_BS_ showed a straighter path probably occurring during inter-patch movements or when transiting from surface to/from depth. Successive broken stick segments of the same behavioural mode_BS_ were then grouped in hunting or transiting phases_BS_ allowing us to quantify the phases_BS_ within each dive (Fig.2 B). For each dive, we characterized each phase_BS_ using the behavioural mode_BS_ (i.e. hunting vs transit), the number of broken stick segments making up each phase_BS_, its duration, its mean depth and its mean ascent/descent rate (Script S1 and Dataset S1). For the SES data set, we also counted the number of prey capture attempts that occurred in each behavioural phase_BS_. They were calculated from the concurrent high resolution acceleration data [51], [52]. Briefly, acceleration data were used to identify rapid head movements that may be associated with prey encounter events and these are visible as spikes in the filtered acceleration profiles [25]. Acceleration profiles with more than one spike above a given threshold (in m/s^2^) visible both in the surge and heave axes were considered to be related to prey encounter events. A full description of the acceleration data filtration process and definition of the threshold for the spike occurrence are given in [25] and [52].

#### 4 Comparison of the two behavioural modes_BS_

In the Weddell seal data set a number of segments_BS_ showed very high vertical ascent/descent rates, which may result from depth measurement errors by the sensor. Davis et al. [42] used a velocity sensor recording swimming speed and observed mean maximum speeds up to 5.1±1 m/s depending on the type of dive and location. We therefore removed dives containing segments_BS_ with ascent/descent rates >7 m/s (23 dives in the Weddell seal dataset). In SES the maximum ascent/descent rates of the broken stick segments was 3.5 m/s, therefore all the SES dives were retained.

We compared the number of prey capture attempts (when available), duration, depth, and ascent and descent rates between the two behavioural modes_BS_ estimated with our method (i.e. hunting vs transit) using unilateral Welch tests on two datasets of 10% of the dives randomly selected for each behaviour. We also compared the time spent in hunting mode_BS_ with the bottom time_CA_ identified in the classical method, using unilateral Welch tests on two datasets of 10% of total dives randomly selected [53] [50]. The Welch test allows comparing samples with different variances. “Unilateral” means that we tested if the mean of one sample was significantly greater than the other one.

## Results

### General diving behaviour

The TDRs recorded the diving behaviour of two southern elephant seals for 72 and 73 days from early November to January 2010 (Table 1). The seals performed 3941 and 4254 dives with an average (mean ± SD) of 53±1 and 56±1 dives per day, respectively (Table 1). The mean maximum dive depths were 511±4 m and 475±4 m with average dive durations of 23±0.01 min and 21±0. 1 min, respectively (Table 1).

**Table 1:** General information on tag transmission and diving behaviour.

	Tag deployment	Tag retrieval	Transmission duration (days)	Number of dives	Number of dives per day	Dive maximum depth (m)	Dive duration (min)
SES 1	2010-10-31	2011-01-21	72	3941	53±1 max: 68	511±4 max: 1260	23±0.01 max: 56
SES 2	2010-01-11	2011-01-15	73	4254	56±1 max: 104	475±4 max: 1296	21±0. 1 max: 50
Weddell seal	2003-02-23	2008-10-20	182	11452	63±24 max: 115	67±54 max: 645	10±6 max: 46

Data are given for two adult female southern elephant seals (SES) captured at Kerguelen Island (49°20′ S, 70°20′ E) and one adult female Weddell seal captured at Dumont d'Urville (66°40′ S, 140°00 E). Both species were fitted with TDRs. Accelerometers were also head-mounted on SES.

The diving behaviour of the Weddell seal was recorded for 182 days from late February to late August 2008 (Table 1). The seal performed 11452 dives deeper than 20 m and longer than one minute with an average of 63±24 dives per day (Table 1). The mean maximum dive depths were 67±54 m with average dive durations of 10±6 min (Table 1).

### Foraging behaviour

#### 1 Comparison between the broken stick analysis and prey capture attempts in SES

Dives included an average of 12±0.02 (max: 16, SES 1), 12±0.02 (max: 17, SES 2) and 12±2 (max: 17, Weddell seal) broken stick segments using the broken stick algorithm. However, the fit of the Gompertz model included in the method did not work for 6% of the SES dives and 4% of the Weddell seal dives which were removed from the dataset. For these dives, the relationship between the mean distance and the number of broken stick points was more linear (Fig. S1). Consequently, the model could not detect an inflexion point, which is necessary for determining the optimal number of broken stick points needed to summarize the dive (Fig. S1). In these cases, the number of broken stick points can be determined subjectively by the user (e.g could be determined to suit the averaged mean distance for all dives).

SES dives were rarely associated with more than 40 prey capture attempts, therefore these dives with >40 prey capture attempts were also removed from the dataset (0.1% of the SES dives). Of the remaining SES dives, there were 1369 dives that were not associated with prey capture attempts (17% of the SES dives) but during which the SES spent 8±13 min in hunting mode_BS_. These dives were, on average, 393±6 m deep, 20±2 min long and characterized by 5±0.05 behavioural phases_BS_. The remaining dives (6814) were associated with an average of 11±0.1 prey capture attempts and on average 9±0.05 min were spent in hunting mode_BS_. Foraging dives (dives with >0 prey capture attempts) were on average 512±3 m deep, 22±0.05 min long and characterized by 7±0.02 behavioural phases_BS_. Dives with prey capture attempts were significantly deeper, longer, more complex (as they were characterized by more behavioural phases_BS_) and more time was spent in hunting mode_BS_ than dives without prey capture attempts (Table 2).

**Table 2:** Comparison of dives with or without prey capture attempts as inferred from acceleration data in southern elephant seals.

	Dives w/o PrCA	Dives w PrCA	t	df	p-value
Depth (m)	394±7	514±7	12	1998	<0.001
Duration (min)	21±0.2	22±0.14	5.3	1765	<0.001
Number of behavioural phases_BS_	5±0.06	7±0.06	23	1998	<0.001
Time spent in hunting mode_BS_ (min)	8±0.15	9±0.1	4	1974	<0.001
					

Duration, depth, complexity (number of behavioural phases_BS_) and time spent in hunting mode_BS_ for 1000 dives randomly selected that are associated (w) or not (w/o) with prey capture attempts (PrCA) were compared using unilateral Welch tests.

**Figure 5:**
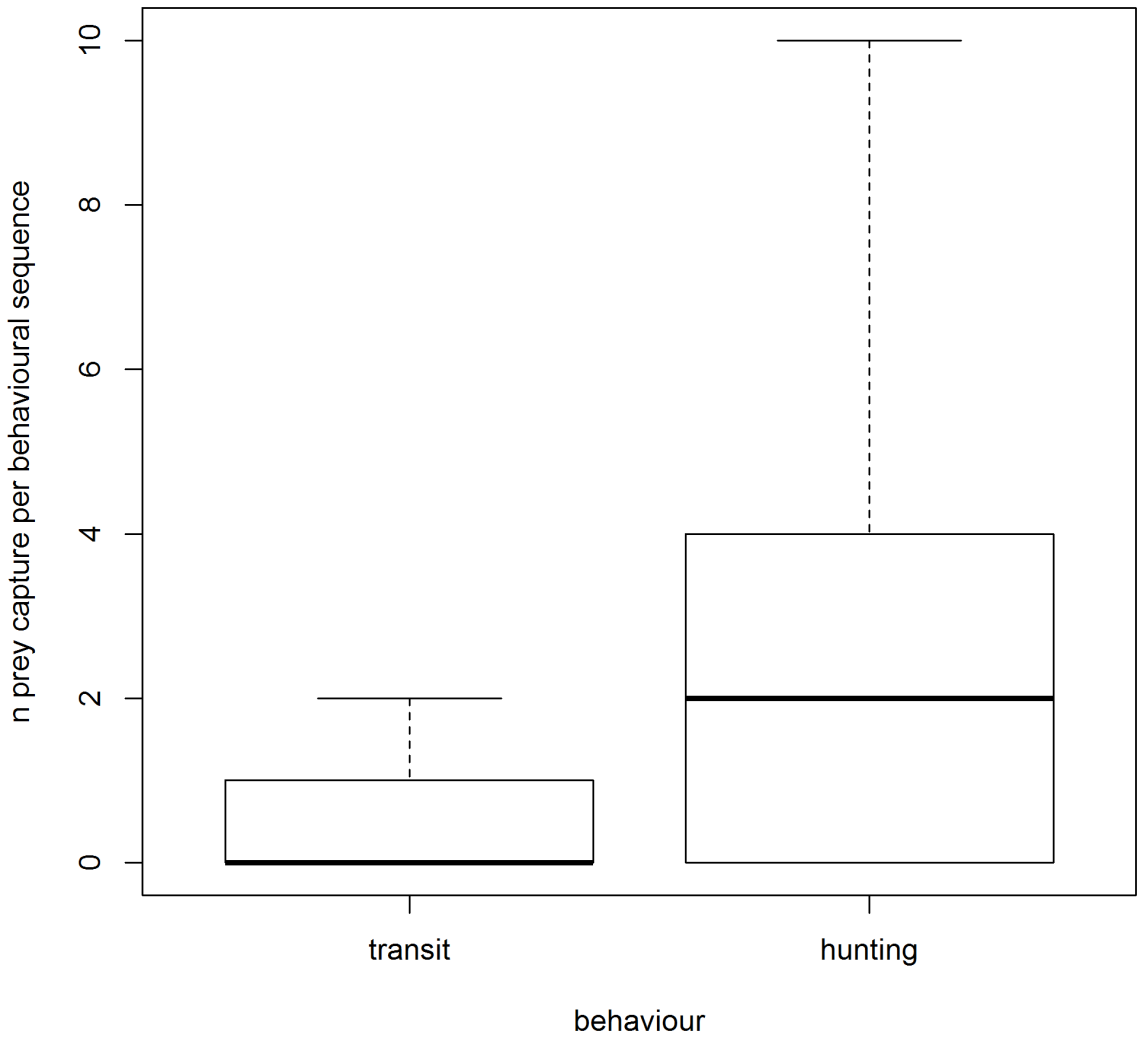
Behavioural differences in prey capture attempts in SES. Distribution of the number of prey capture attempts calculated for each segments_BS_ according to transit mode_BS_ and hunting mode_BS_, respectively for the elephant seal foraging dives.

Hunting phases_BS_ (defined by the broken stick method) of the SES foraging dives were associated with four times more prey capture attempts than transit phases_BS_ (hunting mode_BS_: 2.5±0.02, transit mode_BS_: 0.6±0.007; Table 3, Fig. 5). Of the total prey capture attempts, 77% and 23% occurred during hunting and transit phases_BS_, respectively.

**Table 3:** Comparison of within dive behavioural modes_BS_ in southern elephant seals and Weddell seal.

	Species	Hunting mode_BS_	Transit mode_BS_	t	df	p-value
Depth (m)	SES	386±4	304±3	15	5363	<0.001
	Weddell seal	49±0.9	38±0.6	9.9	5567	<0.001
Duration (min)	SES	2.8±3	2.9±3	1.9	5604	<0.05
	Weddell seal	2.5±3.4	0.9±0.9	27	3314	<0.001
Ascent/descent rate (m.s^−1^)	SES	0.3±0.004	1.23±0.006	126	9153	<0.001
	Weddell seal	0.13±0.001	1.2±0.01	72	7842	<0.001
Number of prey capture attempts	SES	2.5±0.07	0.6±0.02	27	3047	<0.001

Duration, depth, absolute values of ascent and descent rates (mean ± se) and the number of prey captures attempts (SES) between the two foraging modes_BS_ were compared using unilateral Welch tests for two independent sets of 10% of the total dives randomly selected for each modes_BS_. SES stands for southern elephant seals.

#### 2 Comparison of behavioural modes_BS_ defined by the broken stick analysis

**Figure 6:**
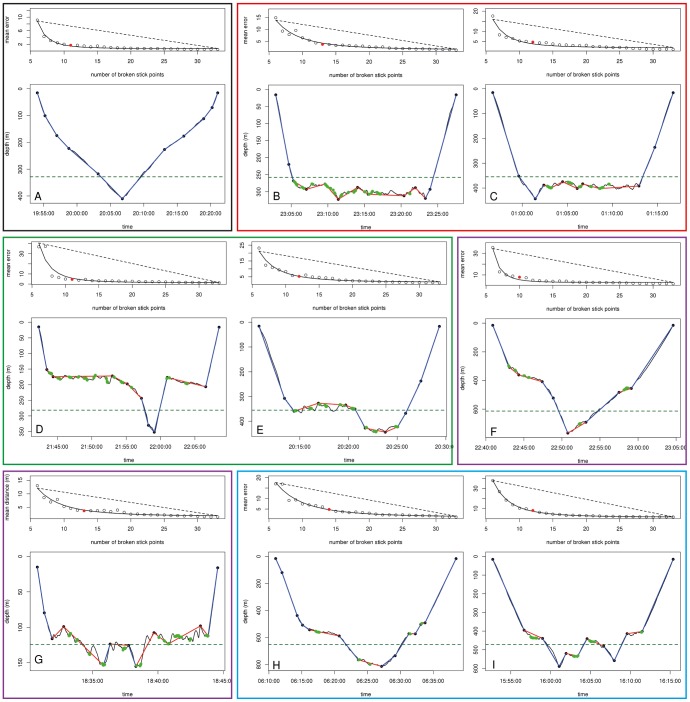
Complexity of the dives for the southern elephant seals. For each panel, the top graph represents the mean distance according to the number of broken stick points in order to select the optimal number of broken stick points to best describe each dive. See figure 2.A for a full description. The lower graph of each panel represents the original dive profile (black line) summarized by the optimal number of broken stick points (black data points). The blue lines represent transit segments_BS_, the red lines represent hunting segments_BS_ and the green dots indicate prey capture attempts (estimated from acceleration data). The green dashed line represents the depth below which bottom time is calculated with the classical dive analysis method. Figures are represented from A to I, from the simplest to the most complex dives, with zero (A, grey frame) to four (H and I, blue frame) hunting phases_BS_.

**Figure 7:**
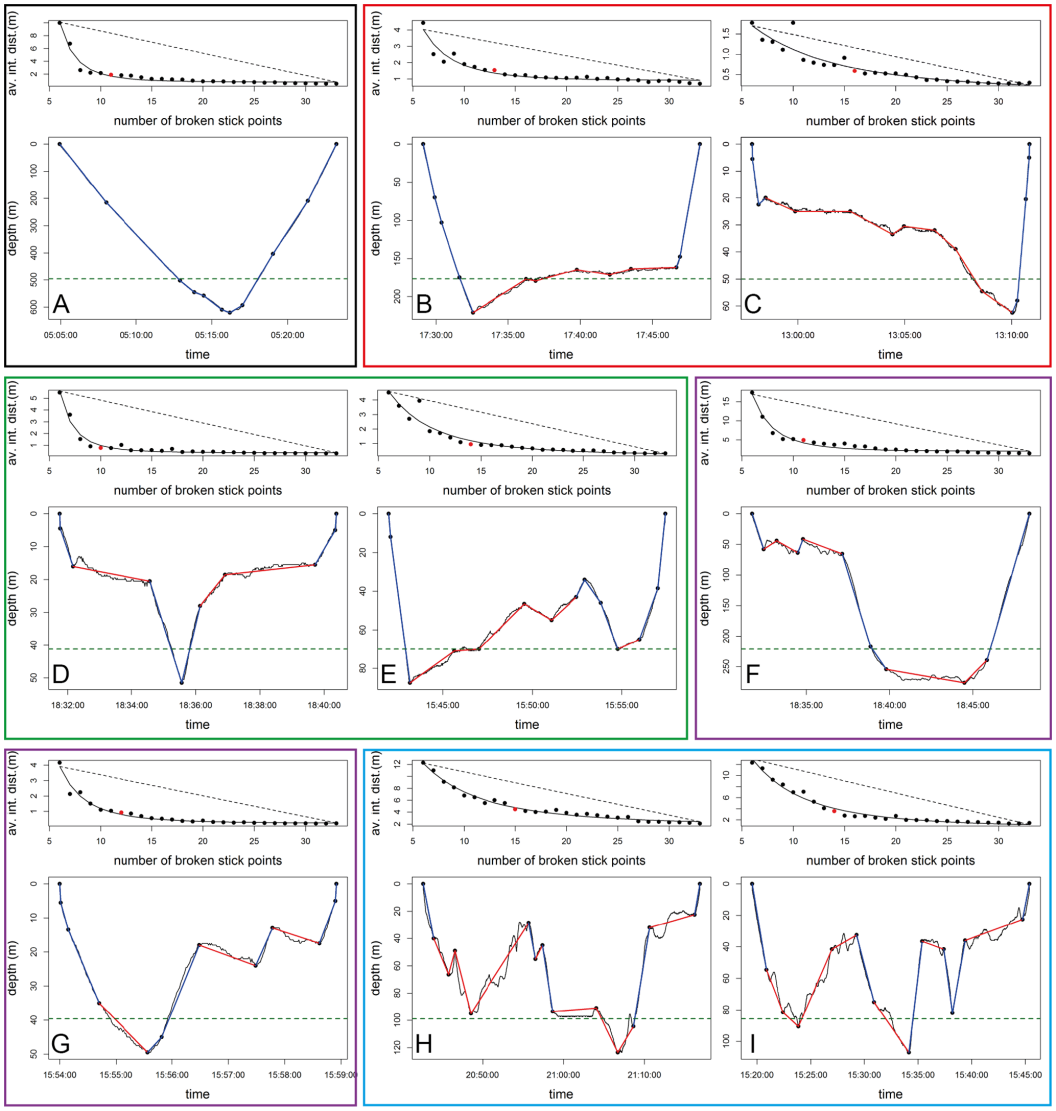
Complexity of the dives for the Weddell seal. For each panel, the top graph represents the mean distance according to the number of broken stick points in order to select the optimal number of broken stick points to best describe each dive. See figure 2.A for a full description. The lower graph of each panel represents the original dive profile (black line) summarized by the optimal number of broken stick points (black data points). The blue lines represent transit segments_BS_ and the red lines represent hunting segments_BS_. The green dashed line represents the depth below which bottom time is calculated with the classical dive analysis method. Figures are represented from A to I, from the simplest to the most complex dives, with zero (A, grey frame) to four (H and I, blue frame) hunting phases_BS_.

**Figure 8:**
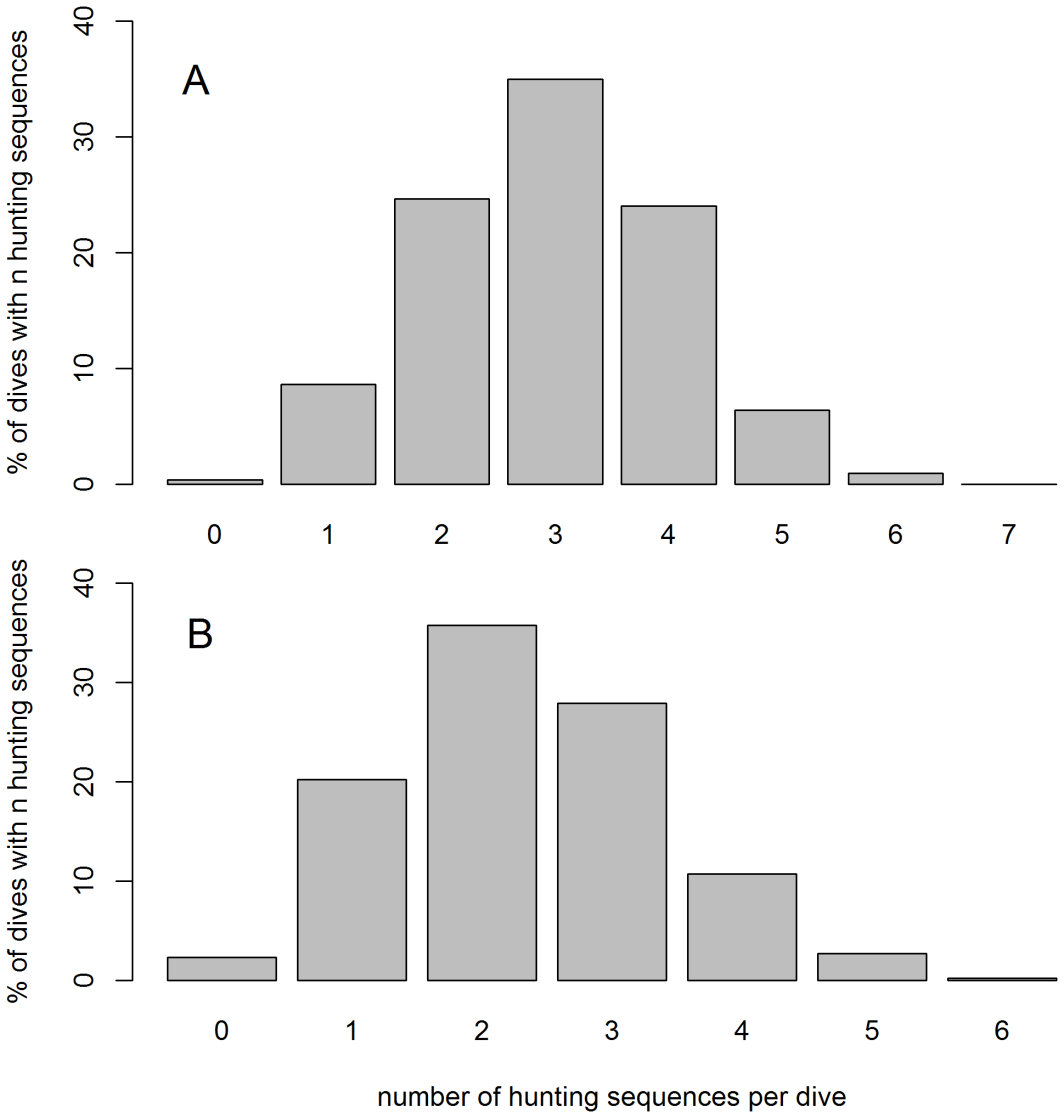
Occurrence of hunting mode_BS_. Proportion of dives containing from zero to seven hunting phases_BS_ (%) for the southern elephant seals (A) and the Weddell seal (B).

Within dive behaviour was characterized by two behavioural modes_BS_: (i) hunting and (ii) transit mode_BS_ (Fig. 1, 6 and 7). On average, dives were summarized by 7±0.03 (max: 15, SES 1), 7±0.03 (max: 13, SES 2) and 6±2 (max: 13, Weddell seal) behavioural phases_BS_. This provides considerably more detail than the simple three phases_CA_ (descent, bottom and ascent phases_CA_) found with the classic dive analysis method (Fig.6 and 7). For the SES, dives with three hunting phases_BS_ were the most frequent (35% of all dives, Fig. 8 a and Fig.6 f-g), followed by those with two (Fig.6 d-e), four (Fig.6 h-i) and one (Fig.6 b-c) hunting phases_BS_ representing, 25%, 24% and 9% of all dives, respectively (Fig. 8 a). Dives with five, six, zero (Fig.8 a) and seven hunting phases_BS_ were scarce, representing 6 to 0.2% of the dives, respectively (Fig. 8 a). Weddell seal's dives with two hunting phases_BS_ were the most frequent (36% of all dives, Fig. 8 b and Fig.7 d-e), followed by those with three (Fig.7 f-g), one (Fig.7 b-c) and four (Fig.7 h-i) hunting phases_BS_ representing, 28%, 20% and 11% of all dives, respectively (Fig. 8 b). Dives with five, zero (Fig.7 a) and six hunting phases_BS_ were scarce, representing 2.7 to 0.2% of the dives, respectively (Fig. 8 b).

**Figure 9:**
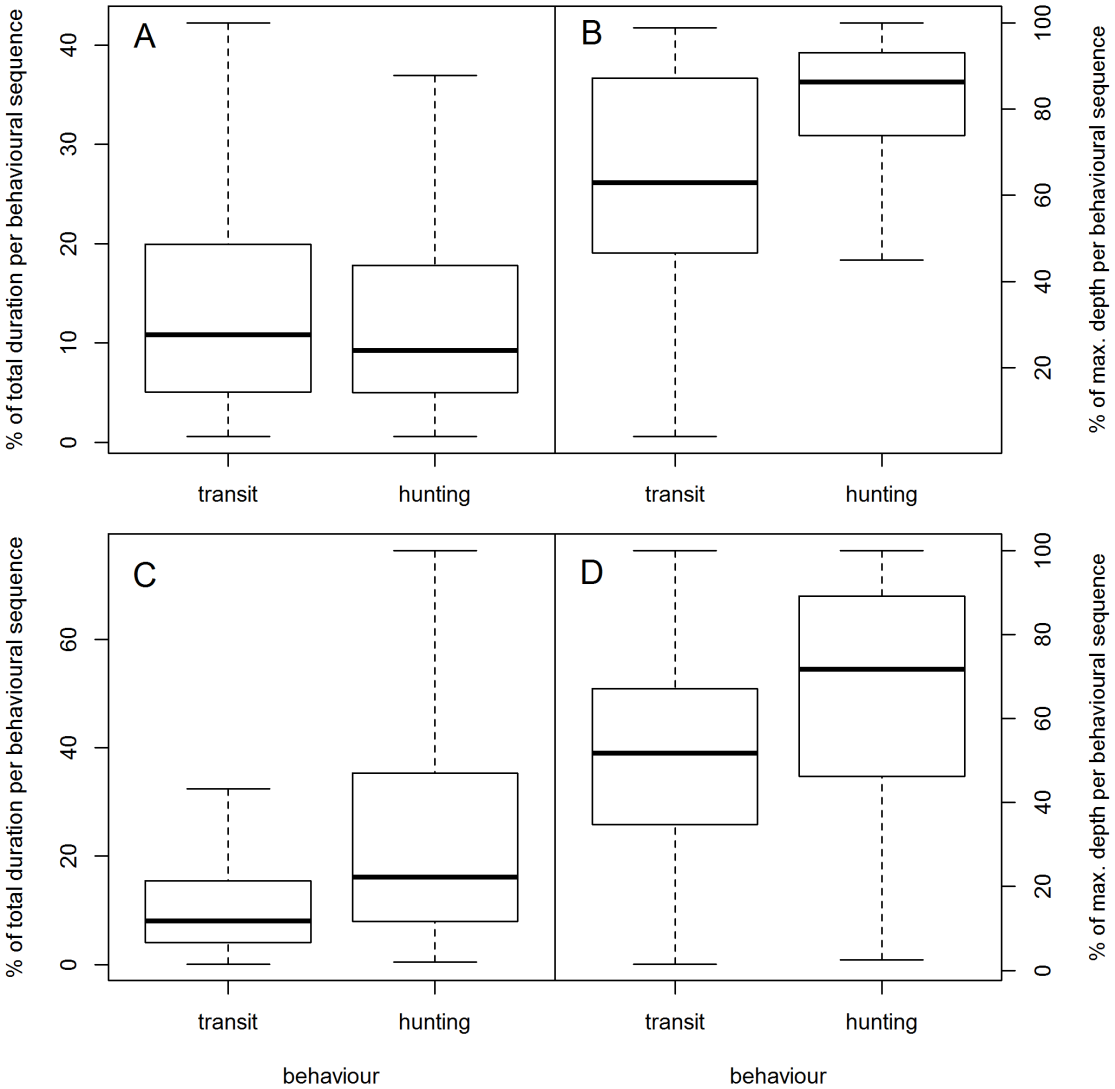
Behavioural mode_BS_ differences. Distribution of each behavioural phase_BS_ duration (sec.) expressed in percentage of the corresponding dive total duration (sec.) for transit mode_BS_ and hunting mode_BS_, respectively (A: southern elephant seals, C: Weddell seal). Distribution of each behavioural phase_BS_ depth (m) expressed in percentage of the corresponding dive maximal depth (m) for each of the two modes_BS_ (B: southern elephant seals, D: Weddell seal). The horizontal bold line of the box shows the median. The bottom and top of the box show the 25^th^ and 75^th^ percentiles.

**Figure 10:**
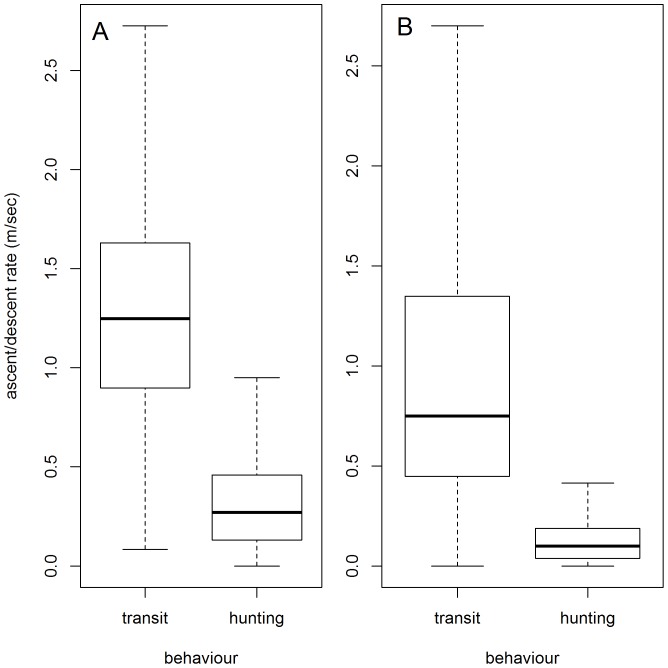
Behavioural differences in ascent/descent rates. Distribution of the ascent/descent rates (m.sec^−1^) calculated for each segments_BS_ according to transit mode_BS_ and hunting mode_BS_, respectively for the southern elephant seals (A) and the Weddell seal (B). The horizontal bold line of the box shows the median. The bottom and top of the box show the 25^th^ and 75^th^ percentiles.

SES hunting phases_BS_ were deeper than transit phases_BS_ as they were localized at 80±0.12% (393±1 m) and 64±0.12% (312±1 m) of the maximal dive depth, respectively (Table 3, Fig. 9 b). Hunting phases_BS_ were shorter than transit phases_BS_ representing 14±0.1% (3±0.01 min) and 15±0.1% (3.3±0.01 min) of the dive duration, respectively (Table 3, Fig. 9 a). When displaying hunting behaviour, SES decreased their instantaneous vertical velocity compared to the one adopted during transit behaviour (hunting mode_BS_: 0.3±0.001, transit mode_BS_: 1.22±0.002; Table 3, Fig. 10 a).

The Weddell seal hunting phases_BS_ were deeper than transit phases_BS_ as they were localized at 66±26% (47±45 m) and 51±23% (36±35 m) of the maximal dive depth, respectively (Table 3, Fig. 9 b). Hunting phases_BS_ were also longer than transit phases_BS_ representing 25±23% (3±3 min) and 12±14% (1±1 min) of the dive duration, respectively (Table 3, Fig. 9 c). The Weddell seal decreased its instantaneous vertical velocity during hunting mode_BS_ compared to the one adopted during transit behaviour (hunting mode_BS_: 0.13±0.13, transit mode_BS_: 1.3±1.9; Table 3, Fig. 10 b).

#### 3 Comparison between the Broken stick and the Classical dive analysis

The SES spent 41% and 59% of their total time foraging when considering the sum of time spent in hunting mode_BS_ and bottom time_CA_ for all dives, respectively. The mean bottom time_CA_ per dive calculated from the classical method was 13±0.05 min whereas time spent in hunting mode_BS_ per dive (i.e. the sum of the different hunting phases_BS_ within a dive) was 9±0.05 min, representing 59±0.2% and 42±0.2% of the corresponding dive duration, respectively. Statistical comparison on 10% of the dives, revealed that bottom time_CA_ was significantly longer than time spent in hunting mode_BS_ (Table 4). The time spent in transit per dive represented 58±0.2% of the corresponding dive duration for the BS method compared to 41±0.2% for the classical approach.

**Table 4:** Comparison of the broken stick and the classical dive analysis.

	Species	Hunting mode_BS_	Bottom time_CA_	n	T	df	p-value
Duration per dive (min)	SES	9±0.07	13±0.08	818	36	7947	<0.001
Duration per dive (min)	Weddell seal	6±0.1	4±0.1	1144	12	2197	<0.001

Duration of the time spent foraging estimated from bottom time (classical dive analysis) and the time spent in hunting mode_BS_ (broken stick method) were compared using unilateral Welch tests for two independent sets of 10% of the total dives selected randomly for both species. SES stands for southern elephant seals.

The Weddell seal spent 67% and 46% of its total time foraging when considering the sum of time spent in hunting mode_BS_ and bottom time_CA_ for all dives, respectively. The mean bottom time_CA_ per dive calculated from the classical method was 4±4 min whereas the time spent in hunting mode_BS_ per dive was 6±5 min, representing 42±26% and 59±25% of the corresponding dive duration, respectively. Unlike the SES, the mean bottom time_CA_ per dive was significantly shorter than the time spent in hunting mode_BS_ per dive (Table 4). The time spent in transit represented 41±24% of the corresponding dive duration for the BS method compared to 58±24% for the classic approach.

In SES 43% of the hunting phases_BS_ occurred above the bottom phase_CA_ identified by the classical approach. For the Weddell seal, 61% hunting phases_BS_ occurred above the bottom phase_CA_ identified by the classical approach (Fig. 7).

## Discussion

In natural systems, predators perceive and react to environmental heterogeneity. These reactions are detected through changes in movement characteristics of animals (*e.g.* direction, speed, sinuosity) [5], [54], that are likely to reflect changes in the presence, or availability, of prey.

We present a new method to quantify the within-dive complexity of diving predators, and demonstrate it using high resolution TDR datasets from two SES and a Weddell seal. We assessed within-dive behavioural phases_BS_ (*e.g.* hunting vs transit) using concepts derived from ARS analyses developed for horizontal track analysis. Our results show: (i) the seals alternated between hunting and transit modes_BS_ at the scale of a dive; (ii) the dives were mainly characterized by numerous behavioural phases_BS_ instead of the three previously described phases_CA_ (descent, bottom and ascent), of which only one (the bottom) was deemed to be involved in foraging; (iii) 77% of total SES actual prey capture attempts occurred in our identified hunting mode_BS_ and intra-dive hunting phases_BS_ were associated on average with four times more prey capture attempts (SES) than transit phases_BS_; (iv) hunting mode_BS_ was adopted two or three times in a dive and was shorter (SES) or longer (Weddell) than that classically estimated from bottom time_CA_. Even though based on a small sample of individuals, this study demonstrates on two seal species that our simple algorithm represents a powerful tool to identify within a dive the parts where the individual intensify its foraging behaviour.

### Detection of intensive foraging activity within dives

Simple depth and time data give a greatly simplified representation of what are very complex and dynamic 3D behaviours. Nonetheless, they still have provided very valuable inferences about key ecological parameters such as foraging, at very relevant temporal and spatial scales [7], [15], [16], [18], [19]. Our approach was based on the transposition of ARS to the vertical dimension. In the horizontal dimension, ARS is characterized by an increase of the trajectory sinuosity and a decrease of displacement speed [5], [6], and is often used as a proxy for intensification of the foraging behaviour [7], [36], [55], [56]. Weimerskirch et al. [57] showed in seabirds, that while food intake could occur outside ARS, it was more predictable in these areas. Here, we identified ARS in the vertical dimension in order to identify those parts of the dive during which the seal increased its foraging activity.

One limit of our study could be that it was based on data from three individuals, though this is compensated to some extent by the very large number of high resolution dives included in the analysis. Nonetheless, two behavioural modes_BS_ were clearly identified in the vertical dimension according to the sinuosity of the dive segments_BS_ identified with the broken stick method.

In our study, 77% of the SES prey capture attempts measured independently occurred during hunting phases_BS_. Acceleration data cannot discriminate between successful prey capture attempts and unsuccessful ones, thus it doesn't give a true estimation of feeding success. Nonetheless it is a proxy for predators interactions with prey [25], [51] and can provide information on the distribution and abundance of prey in the water column [14], [29], [41], [51]. The remaining 23% of the SES prey capture attempts occurred during transit phases_BS_ suggesting opportunistic interactions with more dispersed prey resource [52]. Our results are consistent with transit phases_BS_ representing: (i) transit from the surface to depth of interest or (ii) travel between prey within a dive therefore corresponding to “exploratory phases”. Conversely, the intensification of the seal vertical foraging behaviour can be interpreted as behavioural responses to local increased densities of prey field. During faster, straight transiting parts within the dive, the seal could explore the water column to reach a region occupied by prey. The seal then probably optimizes the time spent in that area by: (i) making “wiggles”; (ii) decreasing its vertical speed and; (iii) horizontally meander at that depth, which cannot be detected with our dataset but which has been previously observed in 3D movements analysis studies [42], [43], [58]. Thus, intensive foraging depths likely correspond to the depths where prey patches are located.

Vertical sinuosity (or wiggles) is often used as an index of foraging effort and/or feeding success even when no independent information on prey capture is available [16], [20], [56], [59]. In our study, non-foraging SES dives were also characterized by some hunting phases_BS_, but they only represented a minority of the dives performed. It is possible that in non-foraging dives SES captured their prey by suction which wouldn't be detected in acceleration data [51]. Feeding by suction has been previously observed for sea lions, leopard, bearded and hooded seals [51], [60]–[62]. Most likely vertical sinuosity is also indicative of searching to locate prey, and therefore still reflects an intensification of the foraging effort [25]. Within foraging dives more prey capture attempts occurred in sinuous phases_BS_ (hunting_BS_). This is in accordance with [41] who showed that intensification of jaw movements during the bottom phase_CA_ of Weddell seal dives were associated with wiggles. Several studies of free-ranging penguins using time-depth data have confirmed that vertical sinuosity was correlated to the occurrence of feeding events measured independently with changes in oesophageal temperature, beak opening events and integrated acceleration-video records [24], [30], [63], [64]. In pinnipeds, vertical sinuosity has also been related to prey capture based on drops in stomach temperature [21]. Furthermore, [65] used video and data recorder to study the 3D dive profiles of Weddell seals in relation to prey encounter and confirmed that vertical sinuosity in time-depth profiles actually occurs during prey encounter.

### Fine scale foraging strategy of Weddell and southern elephant seal

While we are unable to make formal statistical comparisons between the two species due to our sample size, qualitatively we noticed two principal behavioural differences between the SES and Weddell seal: (i) transit phases_BS_ were shorter than hunting phases_BS_ for the Weddell seal whereas they were longer for SES; (ii) hunting phases_BS_ mostly occurred above the bottom phase_CA_ for the Weddell seal whereas they occurred mostly in the bottom phase_CA_ for the SES. These differences probably reflect different foraging strategies between the two species.

Similarly to previous studies, the two SES females essentially used the Antarctic shelf break at sea-ice margin whereas the Weddell seal essentially dived in the fast-iced shallow coastal area in front of Dumont D'Urville [32], [39]. SES performed deeper dives than the Weddell seal and must allocate more time travelling to and from the surface, therefore decreasing the time spent in hunting mode_BS_. Previous studies of Weddell seals using animal borne video and data recorder have shown that the bottom phase_CA_ of dives was associated with significantly higher prey availability than the descent and ascent phase_CA_ [10], [66]. Even though we found that hunting mode_BS_ also occurred during the bottom phase_BS_, it mostly occurred at shallower depths for the Weddell seal. Weddell seals are opportunistic predators feeding both on pelagic prey such as *Pleuragramma antarcticum* and squid, and benthic prey such as *Trematomus* fish species and invertebrates [37], [67], [68]. Their opportunistic behaviour has also been observed during summer where the three dimensional use of the space under the ice by the Weddell seals suggested that they were searching for prey throughout their dive instead of targeting one depth [58].

In contrast, even though we found SES mostly intensified their foraging activity at the bottom of their dive, 43% of their hunting phases_BS_ still occurred above the bottom phase_CA_. This could be related to a more consistent pattern in their foraging strategy due to a more specialized diet. Indeed, SES females essentially perform pelagic dives and a recent study has shown that they were mostly feeding on myctophid fishes [16], [34]. However, our results suggest that considering only the bottom phase_CA_ to fully describe a SES's foraging strategy is probably misleading.

For both species the foraging behaviour revealed by the broken stick was complex. Dives contained on average six or seven behavioural phases_BS_ instead of just three, and hunting mode_BS_ was exhibited on average two and three times a dive, for the Weddell seal and the SES, respectively. Bottom time_CA_ was also significantly higher and lower than hunting time_BS_ for SES and the Weddell seal, respectively, giving a different estimation of the time spent foraging when compared to the time spent hunting_BS_. It is therefore likely that instead of targeting only one type of prey at a particular depth, SES and Weddell seals may also change behaviour mid-dive, to accommodate the sudden appearance of prey. Our novel method allows a more accurate description of the within dive foraging behaviour than when using the bottom time_CA_ only.

## Conclusion

Our study emphasizes the complexity of SES and Weddell seals diving behaviour, suggesting that using bottom time_CA_ only as an index of intensive foraging may lead to an inaccurate estimation of their foraging activity. Our results also suggest that the Weddell seal is an opportunistic feeder capable of chasing prey in different parts of the water column during a single dive whereas the SES mostly increased their foraging effort during the bottom part of their dives. The integration of instrumentation such as video recorders or stomach/oesophageal temperature sensors, from which prey capture success could be inferred, would help validate the method further [21], [24], [30], [42]. This study was based on three individuals of two species but it relies on a broken stick method which detects changes in a dive profile and metrics that can be easily implemented in all diving animals. The consistency observed in foraging strategies across different species [19] suggests that this method could be applied to other species and would be a useful tool to detect behavioural changes when only time-depth data of a sufficient resolution are available.

## Supporting Information

Figure S1 **Examples of dives for which the Gompertz model did not work.** Upper graph: Mean distance according to the number of broken stick points (from 6 to 33) that could be used to describe the dive represented below. The mean distance is the average of the differences between each data point of the original profile and the corresponding point of the reconstructed profile obtained by linear interpolation between the broken stick points (from 6 to 33). Lower graph: original dive profile. Graphs A and B are two examples of SES dive types for which the Gompertz model did not work. For these dives, the relationship between the mean distance and the number of broken stick points was more linear. Consequently, the model could not detect an inflexion point.
(TIFF)

Script S1 **Algorithm of the automated and optimised broken stick method.** R script that allow to apply the broken stick method on high-resolution dives: (i) selection of the optimal number of broken stick points to summarize the dive, (ii) calculation for each broken stick segment of the vertical sinuosity index, descent/ascent rates, duration and depth associated with and (iii) determination of the behavioural mode_BS_ (hunting vs transit) according to the 0.9 vertical sinuosity threshold (see Methods and Fig.4).
(R) Click here for additional data file.

Dataset S1 **Training dataset to run the automated and optimised broken stick algorithm.** Dataset of 1000 dives randomly selected from the Weddell seal dives. Depth was sampled every second by the TDRs during six winter months in 2008 in the Dumont D'Urville coastal area.
(RDATA)
