# Supplementary figures and images for: A New Method to Quantify within Dive Foraging Behaviour in Marine Predators
Source: PLoS One. 2014 Jun 12;9(6):e99329. doi: 10.1371/journal.pone.0099329 (PMC4055756; doi:10.1371/journal.pone.0099329)

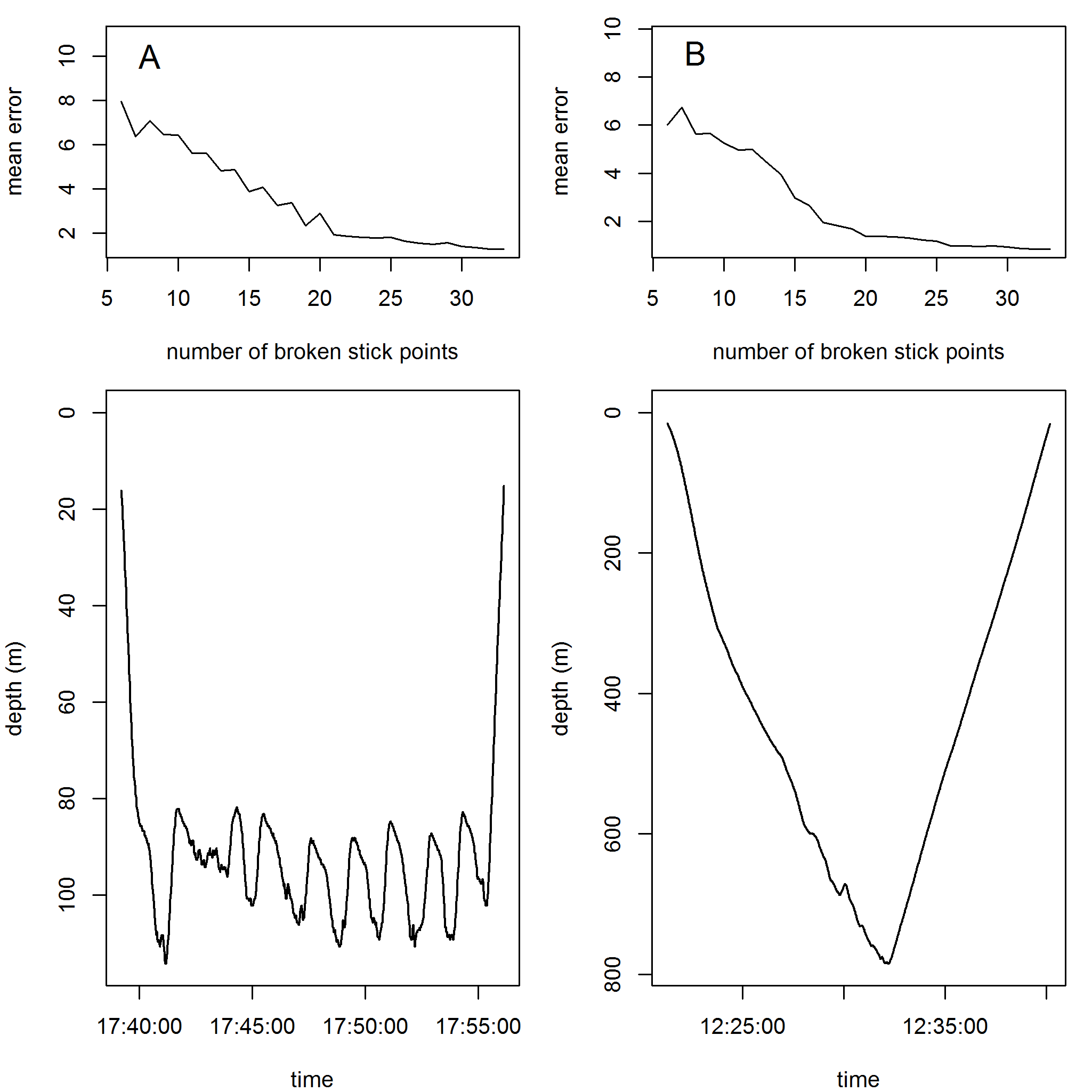

Supplement: Figure S1 — Examples of dives for which the Gompertz model did not work. Upper graph: Mean distance according to the number of broken stick points (from 6 to 33) that could be used to describe the dive represented below. The mean distance is the average of the differences between each data point of the original profile and the corresponding point of the reconstructed profile obtained by linear interpolation between the broken stick points (from 6 to 33). Lower graph: original dive profile. Graphs A and B are two examples of SES dive types for which the Gompertz model did not work. For these dives, the relationship between the mean distance and the number of broken stick points was more linear. Consequently, the model could not detect an inflexion point. (TIFF) [file pone.0099329.s001.tiff]
